# From Donor to Athlete: A Case Study on Post-surgery Return to Sport Following Open Living Liver Donation

**DOI:** 10.7759/cureus.85412

**Published:** 2025-06-05

**Authors:** Connor Farrell, Sarah Manning, Cam Lane, Melissa Novak

**Affiliations:** 1 Sports Medicine, Oregon Health & Science University, Portland, USA; 2 Physical Therapy, Oregon Health & Science University, Portland, USA; 3 Sports Medicine, Portland State University, Portland, USA

**Keywords:** collegiate athlete, musculoskeletal rehabilitation, organ donation, return-to-play protocol, return to sport, soccer player

## Abstract

Living organ donation in high-level athletes is rare, and literature documenting return to sport protocols for these individuals is lacking.

This case report details the experience of a National Collegiate Athletic Association (NCAA) Division I soccer player who underwent living donor hepatectomy and cholecystectomy and documents their return to sport. In this paper, we outline the structured four-phase return to sport protocol that was developed and implemented to support the athlete’s recovery and return to competitive Division I play.

## Introduction

Liver donation is a rare event in the general population and even less documented in elite athletes. Given its complexity, there is potential for many complications to arise in the postoperative period. In open living donors specifically, postoperative incisional hernia, gastric dysfunction, postoperative cholestasis, and biliary tract pathology are all known risks. Studies have demonstrated that most individuals will return to their pre-quality of life by six months [1,2]. Although this timeframe may be appropriate for the general population, it does not take into consideration the specifics of being an elite athlete. Currently, there is limited evidence examining specific return to sport (RTS) protocols for living liver donors. This case details the experience of a collegiate soccer player who underwent open living donor hepatectomy and cholecystectomy. It outlines the structured RTS protocol that was developed and implemented to support the athlete’s recovery and return to competitive Division I play.

This article was previously presented as a poster at the 2024 American Medical Society for Sports Medicine (AMSSM) Annual Meeting in Baltimore, Maryland.

## Case presentation

History

A 21-year-old female collegiate soccer player presented to the training room seeking guidance on her RTS following a living liver donation. One month prior, she had undergone an open living donor hepatectomy of the right lobe and cholecystectomy with prophylactic hernia mesh placement. The surgery was uncomplicated, and she remained under observation for five days postoperatively before being discharged home. During postoperative observation, her alanine aminotransferase (ALT) and aspartate aminotransferase (AST) levels demonstrated a steady decline from 226 U/L (ALT) and 204 U/L (AST) to 102 U/L and 60 U/L, respectively. By her one-month follow-up, her laboratory values had normalized to baseline levels of 55 (ALT) and 31 (AST). Although her general surgeon cleared her to resume activity at that time, no specific guidelines were provided for a return to high-level athletic participation.

Return to sport

Although the athlete’s surgeon cleared her for activity after four weeks and her liver function tests (LFTs) had normalized, devising an RTS plan for elite competition posed significant challenges. The current literature on RTS following intra-abdominal surgeries is limited, and even more scarce for high-level athletes who are organ donors returning to competition. One relatively comparable article was identified, which detailed an RTS protocol for a soccer player who had experienced a bowel perforation and required emergent laparoscopy [3]. This article served as a general framework for developing our protocol, and modifications were made to accommodate the more invasive nature of our athlete’s surgery.

Ultimately, a four-phase RTS protocol (Table 1) with graded progressions was created to ensure a safe return to play. Each phase was guided by the athlete's progress and focused on three key categories: strength, mobility, and conditioning. Each phase included specific restrictions and functional goals. Weekly check-ins with the physical therapy team were conducted to evaluate the athlete’s readiness to advance to the next phase.

**Table 1:** Four-phase RTS protocol with graded progressions RPE: rate of perceived exertion

Phase	Strength Focus	Mobility Focus	Conditioning Focus	Restrictions	Criteria to Progress
Phase I: Pain Control and Soft Tissue Healing	None	Gentle core mobility starting week four	Gentle bike (RPE <3) if fatigue is managed	At least six weeks of bending, lifting, and twisting restrictions of no greater than 10 lbs	Completion of the six-minute walk test and the 30-second sit to stand
Phase II: Restarting Endurance, Mobility, Strength	Re-introduce core and general fitness with weightlifting	Core– increase sets from prior week	Begin jogging (RPE 3-5) and progress as tolerated	Not pushing through fatigue or pain	Able to jog without pain or discomfort
Phase III: Strength, Endurance, Conditioning Building	General fitness - building strength	Core	increased cardiovascular fitness as it relates to sport (run, plyometrics, cutting)	None	Completion of return to run progression without pain or discomfort
Phase IV: Sport-Specific Movements/RTS	Continue building	Maintenance	Implementation of sports-specific activities (ie, ball handling, kicking, dribbling) and rejoin team practice	None	Ability to perform sports-specific activities without pain or discomfort

Phase I of the RTS protocol prioritized pain management and soft tissue healing immediately after and during the weeks following surgery. For the first four weeks, activity was restricted according to the postoperative guidelines provided by the surgeon (Table 2). After this initial period, an additional two weeks of restrictions were implemented, limiting bending, lifting, and twisting to a maximum of 4.5 kg (10 lbs). During this two-week phase, the athlete was permitted to gently bike at low or light effort, provided there was minimal fatigue. For these two weeks, this effort was defined as a rate of perceived exertion (RPE)-an individual's subjective measurement of effort, scaled from 1 to 10 and ranging from 1 to 3.

**Table 2:** Postoperative instructions

Category	Instructions
Diet	Resume regular diet as was directed during your stay.
Activity	Walking is highly encouraged. No lifting greater than eight pounds (e.g., a gallon of milk) or strenuous exercise for six to eight weeks following surgery or until released by a physician.
Wound Care	Keep wounds clean and dry. May shower and let water/soap run over wounds, but avoid scrubbing. No baths, swimming, or submersion until completely healed.
General Instructions	Inspect the incision daily for drainage, increasing tenderness, swelling, or erythema.
Notify Provider If:	Temperature ≥ 38°C (100.4°F) or chills, difficulty breathing, decreased strength/sensation in extremities, uncontrolled bleeding, elevated blood pressure (twice, two hours apart), severe abdominal pain, nausea, vomiting, or diarrhea, painful, bloody, or decreased urination.

Additionally, a light core mobility program was introduced (Table 3), though no strength training was incorporated due to postoperative weight restrictions. Progression to the next phase was contingent upon satisfactory performance in a 30-second sit-to-stand and a 6-minute walk test.

**Table 3:** Core mobility program for phase I and phase II Cat-Cow: see Video 1 for a demonstration Dead Bug: see Video 2 for a demonstration

Exercise	Frequency
Cat-Cow	1 set x 10 reps daily
Quadruped Full Range Thoracic Rotation With Reach	1 set x 10 reps daily
Supine Lower Trunk Rotation	1 set x 10 reps daily
Dead Bug	3 sets x 10 reps daily
Supine Diaphragmatic Breathing With Pelvic Floor Lengthening	1-2 sets x 10 reps daily
Standing Sidebends	1 set x 10 reps daily
Seated Thoracic Flexion and Rotation With Arms	1 set x 10 reps daily

**Video 1:** Cat-cow exercise

**Video 2:** Dead bug exercise

Phase II was focused on reintroducing strength movements, enhancing mobility, and building endurance. Strength training was resumed through body weight exercises and light weightlifting, under the guidance of the strength and conditioning team. Mobility work continued, incorporating the exercises from Phase I, while endurance training advanced to include jogging and light running (Table 4). Restrictions during this phase emphasized monitoring fatigue levels and cessation of activity if abdominal pain or incision changes occurred (Table 5). Progression to the next phase occurred when the athletes’ ability to jog or lightly run at a moderate RPE without excessive fatigue.

**Table 4:** Return to run progression

Week	Days 1 & 2	Days 3 & 4	Days 5 -7
1	6 sets: walk 4 min + run 1 min	6 sets: walk 4 min + run 1 min	6 sets: walk 3 min + run 2 min
2	6 sets: walk 3 min + run 2 min	6 sets: walk 3 min + run 2 min	6 sets: walk 2 min + run 3 min
3	6 sets: walk 2 min + run 3 min	6 sets: walk 1 min + run 4 min	6 sets: walk 1 min + run 4 min
4	Run 30 min	Run 30 min	Run 30 min

**Table 5:** Soreness rules for abdominal pain ATC: certified athletic trainer; PT: physical therapist

Criteria	Action
Soreness during warm-up that continues	Two days off, drop down one step
Soreness during warm-up that goes away	Stay at the same step that caused soreness
Soreness during warm-up that goes away but redevelops during the session	Two days off, drop down one step
Soreness the day after lifting (non-muscle soreness)	One day off, do not advance the program to the next step
No soreness	Advance program one step per week or as instructed by a healthcare provider (ATC, PT)

The goal of Phase III was to build on the foundation established in Phase II by increasing the volume, load, and complexity of strength training and enhancing the intensity of cardiovascular training. This phase incorporated more dynamic and sports-specific movements such as running, cutting, jumping, and plyometric exercises. The core focus and restrictions remained unchanged.

In Phase IV, the athlete transitioned to sports-specific movements and activities tailored to soccer and their playing position. This phase included ball-handling drills, such as dribbling, passing, and kicking, and gradually progressed to full-contact practices. Graduation from the protocol was achieved when the athlete could tolerate these activities for one week without experiencing abdominal pain or excessive fatigue.

Throughout the RTS process, the athlete was closely monitored by a multidisciplinary medical team, including the athletic training staff, physical therapists, and team physicians. Upon completing the protocol, the athlete underwent assessment by the team physicians to evaluate both physical and mental readiness to return to competitive sport.

## Discussion

The current body of literature on RTS for high-level athletes following open intra-abdominal surgery is minimal. Existing research is limited and focuses on RTS after blunt abdominal trauma or injuries sustained during competition or practice, such as liver and splenic injury or bowel perforation [4]. Preplanned surgical events also lack specific RTS information. Generally speaking, most recommendations remain vague and give inconsistent time frames. For example, in a 2021 German survey, 23.9% of surveyed hospitals suggested refraining from sports for six weeks after laparotomy, while 23.4% proposed four weeks, and 15.1% advised only two weeks [5]. Another review based on survey responses of general surgeons recommended avoidance of ball sports, weight training, and athletics for two weeks following laparoscopy and four weeks after laparotomy [6]. Outside of timelines, limited focus exists in the literature about the specifics of what a return to sport looks like. Given this scarcity of data and specifics, we drew on generalized return-to-activity recommendations and protocols from liver injury to inform our athlete’s specific return-to-play process.

For athletes recovering from blunt liver trauma, it is generally recommended that athletes not participate in activity until anatomic and functional healing of the liver is confirmed [4]. This healing process can be monitored through blood tests measuring liver function tests (LFTs) [7]. Reported RTS timelines following liver injury range from two to six months, depending on the extent of the injury [4,8].

Our athlete achieved normalized LFTs at her one-month follow-up, at which point her gradual return to activity was initiated. The complete RTS process took 10 weeks from the date of her surgery, aligning with recovery timelines comparable to those for low-grade liver injuries.

Another key factor that influenced our RTS protocol was the risk of postoperative hernia, a common complication following intra-abdominal surgeries. To mitigate this risk, a prophylactic hernia mesh was placed during the surgery. The athlete’s surgeon cleared her for activity at her four-week follow-up, consistent with biomechanical studies indicating that the abdominal wall demonstrates resistance to strain after four weeks [6]. During her RTS progression, she did not experience any setbacks or complications. This outcome suggests that initiating an increased training load after four weeks may be a suitable benchmark for athletes recovering from open abdominal surgeries to safely begin more intensive activity.

The athlete returned to training with her team 10 weeks post-surgery. However, as the soccer season had concluded, she was unable to participate in competitions and elected to redshirt, formally sitting out the season without affecting her eligibility. Her six-month follow-up lab work demonstrated stable LFTs, suggesting our RTS program did not have a negative effect on her liver function. Additionally, the timeline of her RTS aligned with those reported for other high-level athletes recovering from intra-abdominal surgeries or liver injuries [3,9,10].

While the time frame for this RTS case was similar to others, broad generalizations cannot be made about RTS following abdominal surgeries. Variations in recovery can occur based on specific demands of different sports and even between positions of the same sport. Therefore, RTS protocols must be individualized and tailored to reflect the unique needs and goals of each athlete as they return to their sport and specific position.

## Conclusions

Return to play in high-level athletes following organ donation is extremely rare. When developing RTS protocols for these athletes, it is crucial to consider the type of surgery performed (i.e., open vs. laparoscopic) and the specific organ donated, as these factors will influence the management approach. Additionally, protocols should be tailored to each athlete, incorporating goal-oriented programming and functional testing to ensure a safe and effective return to sport.

Future research should focus on developing sport-specific recommendations and guidelines for RTS following intra-abdominal surgeries in athletes. This would enhance the understanding and management of recovery, supporting athletes in safely resuming high-level competition.
